# Predictors of poor adherence to antidiabetic therapy in patients with type 2 diabetes: a cross-sectional study insight from Ethiopia

**DOI:** 10.1186/s13098-020-00567-7

**Published:** 2020-07-16

**Authors:** Gebre Teklemariam Demoz, Shishay Wahdey, Degena Bahrey, Halefom Kahsay, Gebremariam Woldu, Yirga Legesse Niriayo, Andrew Collier

**Affiliations:** 1 School of Pharmacy, College of Health Sciences, Aksum University, PO.Box: 298, Aksum, Ethiopia; 2 School of Public Health, College of Health Sciences, Mekelle University, Mekelle, Ethiopia; 3 School of Nursing, College of Health Sciences, Aksum University, Aksum, Ethiopia; 4 School of Pharmacy, College of Medicine and Health Sciences, Adigrat University, Adigrat, Ethiopia; 5 School of Pharmacy, College of Health Sciences, Mekelle University, Mekelle, Ethiopia; 6 Department of General Medicine, University Hospital Ayr, Ayr, KA6 6DX Scotland, UK

**Keywords:** Type 2 diabetes, Predictors, Medication non-adherence, Antidiabetic medication, Ethiopia

## Abstract

**Background:**

Poor adherence to the medical regimen is a major clinical problem in the management of patients with diabetes. This study sought to investigate the level of medication adherence to antidiabetic therapy and to identify possible predictors of poor adherence.

**Methods:**

A hospital based cross-sectional study was conducted from July 2018 to June 2019 among randomly selected follow-up T2D patients at a hospital diabetes clinic. Data were collected through patient interviews, followed by medical chart review. Adherence to antidiabetic therapy that we assessed patients’ responses using validated Brief Medication Questionnaire (BMQ). To identify predictors of poor medication adherence, binary logistic regression analyses were performed using SPSS version 25. Statistical significance was set at *p* value ≤ 0.05.

**Results:**

Of the total 357 study participants, 25% were non-adherent to their antidiabetic therapy. Predictors statistically associated with poor adherence were; being female gender (AOR = 1.71, 95% CI 1.01–2.76), and presence of at least one diabetic complication (AOR = 2.02, 95% CI 1.02–3.22). Participants with having at least primary level of education were more likely to adhere to anti-diabetes medication (AOR = 0.42, 95% CI 0.18–0.96). The most common self-reported reasons for non-adherence were forgetfulness, unavailability of medication plus the unaffordability of anti-diabetes medications.

**Conclusions:**

The proportion of participants’ adherent to anti-diabetes therapies was suboptimal. Being female, the presence of chronic diabetic complications and having no formal education were the main predictors of poor adherence. Strategies that aimed at improving adherence to antidiabetic medications deemed to be compulsory.

## Background

Diabetes is a growing global public health problem with an estimated 425 million people affected in 2018 and is projected to rise to 629 million by 2040 with increases particularly prevalent in developing nations. The prevalence of people living with diabetes in Ethiopia is substantially increasing annually [1, 2]. According to World Health Organization (WHO) therapy adherence is a primary contributing factor for treatment success. Non-adherence to medications regimens reduces optimum clinical welfare and outcome [3].

Poor medication adherence leads to increased health care resource costs, including more frequent hospitalizations [4]. Non-adherence to the medical regimen is a major clinical problem in the management of patients with diabetes is a global problem. In developed nations approximately 50% of diabetic patients do not adhere to the recommended therapies. While, this situation is far worse than in the developing world [3]. Particularly, non-adherence to medication is common in patients with diabetes [5] and non-adherence compromises safety and treatment effectiveness, leading to increased morbidity and mortality rates. This contributes to significant direct and indirect costs in the healthcare system [6–8].

Several studies have identified the predictors of poor adherence to anti-diabetes medications [8–10]. Socio-demographics included age, sex, ethnicity, income, education, and clinically-related conditions were often found to be predictors of poor adherence [5, 11, 12]. In Ethiopia the rate of poor adherence to anti-diabetes medication is prevalent [13] and poor adherence is a predictor for poor glycemic control [14]. The most common reasons for poor adherence include patients not being conscientious with their regimens, patients forgetting to take their medications regularly, and patients stopping when they feel better or worse [15, 16]. Drug side effects, regimen complexity, failure to remember, low grade of education, lack of transportation, low monthly income, and absence of a home glucometer were also reported as predictors that commonly accounted for poor adherence to anti-diabetes medication [17, 18].

Diabetes care is complex and requires multidisciplinary medical care plus patient self-management and education [19, 20]. Improved adherence levels may improve overall health care resource utilization and costs [4]. In contrast, poor adherence rates may contribute to problems related to health care utilization and increases diabetes burden to the community and government. In addition non-adherence to long-term therapies may lead to poor treatment outcomes and increased health care costs. Because of limited number of studies that have been conducted in Ethiopia, there is a gap in the knowledge in predictors for non-adherence to anti-diabetes therapies. Unfortunately, there have been previous studies that the level of adherence is well known, but few studies have investigated the potential predictors of poor adherence so as level adherence is still not improved that require intervention. Hence, understanding the potential predictors of poor medication adherence is imperative for ensuring that interventions are developed and implemented to support adherence to diabetic therapies where identifying which patients have truly affected by which predictors of medication non-adherence is important before additional therapies or other interventions are introduced. This study adds to our knowledge of the possible predictors of non-adherence in this important group of patients with diabetes. This may be followed by involving successfully in addressing such predictors once identified to improve adherence and patient treatment outcomes and how these efforts can effectively be incorporated into clinical care for patients with diabetes is required. Therefore, our study was aimed to describe the therapeutic adherence and to explore the frequency of individual reasons for poor adherence plus identifies significant predictors of poor adherence to antidiabetic therapies in Ethiopia so that there can be suggested some strategies to plan interventions.

## Methods

### Study participants

A hospital based cross-sectional study was undertaken from June 2018 through July 2019. Aksum University referral hospital is the second largest public referral and teaching hospital in northern Ethiopia and is affiliated with Aksum University. Participants recruited in this study had type 2 diabetes, aged 18 years and above, ambulatory, and were taking at least one anti-diabetes drug. Patients had to be attending the diabetes clinic for longer than 3 months. The sample size required was calculated using the formula.

$$ n = \frac{{\left( {Z_{{{\raise0.7ex\hbox{$\alpha $} \!\mathord{\left/ {\vphantom {\alpha 2}}\right.\kern-0pt} \!\lower0.7ex\hbox{$2$}}}} } \right)^{2} P\left( {1 - P} \right)}}{{d^{2} }} $$

(where n = required initial sample size, *Z* 𝑎/2 = critical value for normal distribution at 95% confidence interval which equals 1.96 (𝑍 value at alpha = 0.05), 𝑃 = proportion of success; (p = 0.5), 𝑞 = proportion of patients with diabetes good adherence (*q *= 0.5), and 𝑑 = marginal error (5% = 0.05). Thus, we calculated the minimum sample size of participants using a single population formula, by taking 50% of population and found 384. A simple random sampling technique was used based on patients’ sitting for their regular follow-up, whereby participants were recruited until the predetermined sample size was achieved in the consecutive appointment days. Out of these, 357 patients were included in our final analysis that 27 subjects were excluded because of participants’ chart were found to be with incomplete record.

### Variables

The variables included in this study were age, gender, marital status, social habits; physical activities, smoking, alcohol consumpition, clinical and disease related conditions, presence of co-morbid conditions and diabetes complication, body mass index (BMI), glycemic control, duration of diabetes, drug therapy, diabetes related hospitalization, source of medication, availability and affordability issue of antidiabetic medications, experiencing side effects, use of alternative medicine plus residence (rural or urban). Level of education was based no formal education (unable and/or able to read and/or write), primary (grade 1 to 8), secondary (grade 9 to 12), tertiary (college diploma and above). Level of medication adherence to antidiabetic agents was our dependent variable.

### Data collection tolls and procedures

Data was collected using structured questionnaire which involved face to face interviews by trained interviewers. In our study, basically we focused on medication adherence. A full explanation on the purpose of the study was provided for each participant, after which the participants gave their written informed consent. Interviewers asked the participants about the duration of anti-diabetes drug therapy and follow up, to ensure that they were on antidiabetic drug therapy for at least 3 months and had regular follow up. Data were collected in two phases. Phase 1: primary data (age, gender, marital status, educational status, adherence to physical activities, smoking atatus, residence, source of medication, alcohol consumpition, BMI, drug side effects, use of traditional medicine, medication adherence and possible reasons for non-adherence) were collected from patients face to face using interviewer administered interview. Interviewers asked the participants about the duration of anti-diabetes drug therapy and follow up, to ensure that they were on antidiabetic drug therapy for at least 3 months and had regular follow up. Phase 2: secondary data (glycemic control status and other laboratory values, diabetes-related complication, comorbid conditions, diabetes-related hospitalization history, prescribed medications and related issues) were collected from medical chart.

Adherence to antidiabetic therapy was assessed using validated Brief Medication Questionnaire (BMQ) [21] and determined through self-reports of what medications had been taking over the week prior to the interview. BMQ is a self-reporting tool used to identify patients’ poor adherence and barriers to adherence. In our study we used BMQ tool to assess adherence to antidiabetic therapy [22]. The BMQ tool includes a 5-item regimen screen that asks patients how they took each medication in the past week, a 2-item belief screen that asks about drug effects and bothersome features, and a 2-item recall screen about potential difficulties remembering. Participants were specifically asked to recall possible reasons if they missed any doses of medication on a day to day basis or over a period of 7 days.

### Data processing, analysis and presentation

Database was set and entered using EpiData Manager Version 4.0.2.00, EpiData Entry Client, respectively (EpiData Association, Denmark). Then, data were exported into SPSS version 22.0 **(**SSPS Inc., Chicago, IL, USA) for analysis. Descriptive statistics included mean ± standard deviation (SD) for normally distributed continuous variables and frequency and percentage for categorical data were used to summarize socio-demographic and relevant clinical characteristics of the study participants. Binary logistic regression model analysis was used to investigate associations and possible predictors of poor medication adherence. Variables in bivariate analyses with p-value ≤ 0.20 were further analyzed in multivariate logistic regression to control the effect of confounders. The final results for the predictors of poor medication adherence were presented using Adjusted Odds Ratios (AORs) with its 95% confidence intervals (CIs). Statistical significance was set at p-value ≤ 0.05.

## Results

### Socio-demographic and clinical characteristics

In this study 357 study participants were included, of them 54.6% were female. The mean (± SD) age of the study participants was 59.4 ± 13.1 years and more than half, 188 (52.7%) of them were in their middle age (41–60) years. Nearly two-fifth (39.5%) of the participants had at least College and above level of education (Table 1). The mean duration of the diabetes was 11.2 ± 8.9 years. The most common comorbid diseases were hypertension, 188 (52.7%), followed by dyslipidemia 171 (47.9%) and ischemic heart disease 37 (10.4%). In addition, the most commonly encountered chronic diabetes-specific complications were diabetic neuropathy 87 (24.4%), followed by diabetic retinopathy 25 (7.0%) and diabetic nephropathy 18 (5.0%). The overall mean (± SD) value of FBG for the previous three consecutive visits was 172.60 ± 44.0 mg/dL. Only 62 (17.4%) of the participants met the intended glycemic target (FBG = 70–130 mg/dL). The mean BMI (± SD) of the participants was 27.15 ± 4.46 kg/m^2^. 76% of the obese (≥ 30 kg/m^2^) participants were female.

**Table 1:** Socio-demographic and clinical characteristics of patients with T2D on follow-up in Ethiopia, 2019

Variables	Categories	Study participants (N = 357)
		Frequency (%)
Sex	Male	162 (45.4)
	Female	195 (54.6)
Age (years)	Mean ± SD	59.4 ± 13.1
	20–40	22 (6.2)
	41–60	188 (52.7)
	> 60	147 (41.1)
Marital status	Single	13 (3.6)
	Married	308 (86.2)
	Divorced	20 (5.6)
	Widowed	16 (4.5)
Religious	Orthodox	294 (82.3)
	Muslim	36 (10.1)
	Protestant	27 (7.6)
Residence	Rural	304 (85.2)
	Urban	53 (14.8)
Level of education	No formal education	50 (14.0)
	Primary (1–8)	63 (17.6)
	Secondary (9–12)	103 (28.9)
	College and above	141 (39.5)
Employment status	Employed	201 (56.3)
	Unemployed	156 (43.7)
Smoking habit	Current smoker	2 (0.6)
	Ex/never smoker	355 (99.2)
Regular alcohol use	Yes	40 (11.2)
	No	317 (87.8)
Adherence to regular physical activity	Yes	252 (70.6)
	No	105 (29.4)
Duration of diabetes (years)	Mean ± SD	11.64 ± 6.95
	1–5	67 (18.8)
	5–10	105 (29.40
	11–15	95 (26.6)
	> 15	90 (25.2)
BMI (kg/m^2^), (n = 71)	Mean ± SD	27.15 ± 4.46
Average of FBG (mg/dL)	Mean ± SD	172.60 ± 44.
Poor glycemic control	< 70 or > 130	295 (82.6)
Good glycemic control	70–130	62 (17.4)
Presence of co morbidities	Yes	278 (77.9)
	No	79 (22.1)
	Hypertension	188 (52.7)
	Dyslipidemia	171 (47.9)
	IHD	37 (10.4)
	Others*	50 (14.0)
Number of comorbidities per patient	Mean ± SD	1.66 ± 0.66
	1–2	252 (70.6)
	≥ 3	26 (7.3)
Presence of diabetes complication	Yes	115 (32.2)
	No	242 (67.8)
	Neuropathy	87 (24.4)
	Retinopathy	25 (7.0)
	Nephropathy	18 (5.0)
Number of complications per patient	Mean ± SD	1.22 ± 0.53
	1–2	108 (30.3)
	≥ 3	5 (1.4)
Level of adherence	Adherent	268 (75.0)
	Non-adherent	89 (25.0)

Others*: Asthma and Thyroid disorders. *FBG* Fasting Blood Glucose, *IHD* Ischemic heart disease, *SMBG* Self-Monitoring of Blood Glucose

### Medication adherence to antidiabetics and reasons for non-adherence

This study was intended to measure the level of non-adherence. Medication adherence was grouped as non-adherence and adherent. 25% of participants were reported as non-adherent to their medication. The most common self-reported reason for non-adherence reported by participants was unintentionally forgetting (36.7%) to take of their medicine, followed by inadequate availability of medications (28.6%) and lack of affordability (12.6%) (Table 2).

**Table 2:** Reasons for medication non-adherence among patients with T2D on follow up in Ethiopia, 2019

Reasons	Study participants (N = 357)
	Frequency (%)
Forgetting to take their medicine	131 (36.7)
Inadequate availability of medication	102 (28.6)
Cost of medication too expensive	45 (12.6)
Fear of medication adverse events	35 (9.8)
Difficulty of administration/time schedule	28 (7.8)
Inadequate instruction/counseling/education	26 (7.3)
When feeling better or worse their disease	23 (6.4)
Busy due to workload	13 (3.6)
During fasting period	21 (5.9)
Regimen complexity of the medicine	21 (3.6)
Patient prefers not to take the medicine	15 (2.8)
Disbelief in medicine effectiveness	13 (3.6)

Percentages are calculated per column

### Predictors of poor medication adherence

As illustrated in Table 3, the associated factors statistically significant with poor medication adherence in the multivariable analysis were gender, level of education and diabetes complication. From the AOR, being female gender (AOR = 1.71, 95% CI 1.01–2.76, p = 0.047) was positively associated with poor adherence to their anti-diabetes medication. In addition participants who had completed primary, secondary and college and above level of education (AOR = 0.38, 95% CI 0.21–0.92, p = 0.043, AOR = 0.42, 95% CI 0.19–0.90, p = 0.023), and AOR = 0.39, 95% CI 0.29–0.95, p = 0.041), respectively were less likely to poorly adhere to their medication regimen. However, the presence of at least one diabetes complication (AOR = 2.02, 95% CI 1.01–3.22, p = 0.008) reduced the likelihood of adherence to the medication regimen.

**Table 3:** Bivariate and multivariable analysis of possible predictors for medication non-adherence in patients with T2D on follow up in Ethiopia, 2019

Covariates	Subcategories	Adherence level		Odds Ratio (95% CI)		P-value
		Adherent, n (%)	Non-adherent, n (%)	Crude	Adjusted	
Sex	Male	135 (79.8)	34 (20.2)	1.00		
	Female	133 (70.9)	55 (29.1)	1.71 (0.97–2.41)	1.57 (1.01–2.76)	*0.047*
Age	20–60	184 (77.6)	53 (22.4)	1.00		
	60+	84 (70.0)	36 (30.0)	1.61 (1.04–2.41)*	1.22 (0.70–2.04)	0.210
Education	No formal education	30 (60.0.)	20 (40.0)	1.00		
	Primary (grade 1–8)	49 (77.8)	14 (22.2)	0.43 (0.20–0.94)*	0.38 (0.21–0.92)	*0.043*
	Secondary (grade 9–12)	83 (80.6)	20 (19.4)	0.40 (0.17–0.78)*	0.42 (0.19–0.90)	*0.023*
	College and above	106 (75.2)	35 (24.8)	0.39 (0.19–0.80)*	0.39 (0.29–0.95)	*0.041*
Employment	Employed	156 (77.6)	45 (22.4)	1.00		
	Unemployed	112 (71.8)	44 (28.2)	0.69 (0.43–1.03)	0.82 (0.61–1.80)	0.610
Complication	Absent	191 (78.9)	51 (21.1)	1.00		
	Present	77 (67.0)	38 (33.0)	1.07 (0.21–0.88)*	2.00 (1.00–3.22)	*0.008*
Source of drug	Free	159 (72.3)	61 (27.7)	1.00		
	Paid	109 (79.6)	28 (20.4)	0.59 (0.39–1.01)	1.34 (0.81–2.19)	0.11
Glycemic control	Poor	219 (74.2)	76 (25.8)	1.00		
	Good	49 (79.1)	13 (28.9)	2.82 (0.90–8.03)*	3.51 (0.91–7.21)	0.141

Percentages are calculated per row. *Variables in bivariate analysis ≤ 0.05, statistically significant set at: p ≤ 0.05

## Discussion

The main finding of this study was that one-fourth (25%) of the study participants adhered poorly to their antidiabetic medications. The predictors independently associated with poor adherence were being female, presence of diabetes complication and having formal education. Results of the present study was in agreement with the previously conducted study in India [23], and two studies conducted in Ethiopia [16, 24]. A higher prevalence of non-adherence rate to antidiabetic medications was reported in a study conducted in Uganda [25]. In our study, a higher percentage of non-adherence rate was observed compared to a further study conducted in Uganda [26]. This variation probably reflects study participants, difference in interview tool, sample size and comorbid conditions. The nature of self-reported medication adherence assessment is open to criticism for a number of reasons particularly the over- or under-estimation of medication adherence.

Meanwhile, the cost or lack of affordability medications also influences diabetic care and thus affects adherence rate of patients [9, 13, 27]. In our study 61.6% of participants received their anti-diabetes medications for free and the costs of antidiabetic medications reported by participants as an important reason in preventing optimal adherence was only 12.6%. This has been supported by studies elsewhere in which financial constraint was recognized as an interference to medication adherence in patients with T2D [13, 23, 27]. For those patients who had authorision, our hospital arranged for a few free anti-diabetes medications (insulin, glibenclamide and glimepiride) to be provided by the hospital diabetes center. However, this was no guarantee to adherence due to intermittent unavailability of these medications.

The incidence of such unavailability and/or interruptions of antidiabetic medications were not uncommon. 28.6% of participants claimed that hospital unavailability was a reason for non-adherence to their anti-diabetes medications. This forced participants to visit the diabetes center repeatedly before their regular appointment or made them purchase their medications from private pharmacies. This led to many patients going without medication or making their purchased their medications last longer. Such irregular availability and/or interruptions of anti-diabetes medications was associated with low aid and supply problems in the health care systems [28, 29]. In addition, longer appointment for follow-up of participants in the center may be associated with prescriptions being obtained from private pharmacies at higher prices. Consequently, this will also negatively impact on their adherence. It is critical therefore that efforts be put in place at diabetes clinic to provide adequate drug supply with the updated diabetes care guidelines and a willingness to arrange appropriate review times with patients [30, 31].

In the current study, factors independently associated with non-adherence were also identified. From the multivariate model analysis, being female gender, participants who had completed at least primary level of education and participants who developed at least one diabetes complication were predictors significantly associated with medication non-adherence. Thus, the present study revealed that, being female gender were more likely non-adherent to their medication than male participants. This result was in agreement with the study conducted by Sajith et al. [23] and Kalyango et al. [25]. Poor self-care were also reported more in female patients with diabetes [32]. Unlike the studies reported by Wabe et al. [13] and Kassahun et al. [17], in our study age and drug adverse events were not statistically significantly in association with medication non-adherence rate.

Previous studies reported that, the most common reasons for non-adherence of their medication were unavailability and unaffordability due to the local cost of antidiabetic medications, fear of drug side effects, regimen complexity, inconvenience of dosage times plus inadequate provision of instruction and education [13, 15–17, 23, 33, 34]. In this study, there was no statistically significant association with participants on multiple medications regimen; drug related adverse effects or presence of comorbid conditions. This was in contrast to the study from Teklay et al. [35], that indicated that related adverse effects and comorbid conditions were associated with medication non-adherence.

In terms of educational status, having primary or secondary level of education decreased the odds of participants being non-adherent to their antidiabetic medication by 58%. This might be explained by the participants ability to read and understand the importance of their adherence to their medication and is consistent with studies conducted in different settings of Ethiopia by Kassahun et al. [17], Jemal et al. [15], and Ali et al. [36] and one study conducted in India by Sajith et al. [23]. In fact, in our study above 60% of participants had completed at least secondary level of education. In short patients with no formal education should be given more information and a better understanding of their medication.

In addition, participants who developed at least one diabetes complication significantly reduced the odds of adhering to their medications (AOR = 2.00). Diabetes complications reduce patients’ adherence rate to their antidiabetic medications by 100% and it could be that once patients develop complications of diabetes they are less inclined to believe that the anti-diabetes tablets are as effective. 32.2% of the participants had developed diabetes complications. Developing diabetes complication is prevalent in Ethiopia [37] and could be due to delayed diagnosing and poor screening for diabetes [38]. The current finding was consistent with else finding reported from Jimma [35], which indicated presence of diabetes complication was a contributing factor for non- adherence in patients with diabetes.

Furthermore, in our study, medication non-adherence was found to be linked with suboptimal glycemic control. This finding was supported by another three studies [39–41]. In this study, though more than three-fourth (76.8%) of participants had an access to use home glucometer for their glucose monitoring, only 17.4% of participants were to be found in the intended glucose target (70–130 mg/dL).This could reflect that patients have poor knowledge about their illness, medications and poor provision of counseling and education service. These points are debatable. Health care providers are working under enormous of pressure and finding further time to support their patients in creating awareness and understanding of their illness and prescribed medications will be difficult.

However, medication non-adherence is not the patient’s problem alone. It might arise from frustration and upset from developing diabetes, plus failure to provide continuous support that the patient needs once the drug has been dispensed. Furthermore, prescribers may fail to increase drug therapy when individuals develop poorer glycemic control. In addition, elevated FBG levels might be linked with poor medication adherence due to the belief that the medication is not working. Hence, there is a need to establish patients’ perspective in ensuring and inspiring discussion about the aim of their drug therapy to resolve such problems related to medication adherence. A lack of achieving glycemic control might also be explained by missing of the correct drug therapy, although 26.1% of participants were receiving two or more anti-diabetes agents at their recent visit. Thus, this diabetic care practice study has shown that, there is a need to improve adherence rate in achieving individualized glycemic targets.

Moreover, findings of the present study demonstrated that anti-diabetes medication non-adherence poses an extensive challenge in patients’ attempts to achieve the desired level of glycaemic control among patients with T2D. Consequently, this work signals a need for continuous comprehensive adherence monitoring and provision of counseling or education service for patients with diabetes particularly for that female gender, and participants developed chronic diabetes complications.

## Conclusions

Findings this study indicated that, the rate of participants with non-adherent to antidiabetic therapy was considerably high. Medication non-adherence was significantly associated with female gender, no formal education and diabetes complication. Therefore, strategies that aimed at improving adherence to antidiabetic medications, drug availability with possible low cost price and provision of medication related counseling could improve adherence. Hence, authors recommend conducting patient-tailored interventional study with a stronger commitment approach is looked-for.

### Limitations of the study

In the present study certain limitations should be considered. Information relevant to our study may be missed or inaccurately recorded as they documented for another purpose. Being the study conducted in a single center was another limitation of the study that the use of a multi-center study would have improved the generalizability of the findings. Self-reported assessment approach of subjects may affect our outcome. We may also be missed some lifestyle changes of participants like diet modification and weight reduction.

## Data Availability

Data that aid the findings of the current study are available from the corresponding author.

## Abbreviations

ADA: American Diabetes Association
CVD: Cardio Vascular Disease
eGFR: Estimated Glomerular Filtration Rate
FBG: Fasting Blood Glucose
IDF: International Diabetes Federation
OGLD: Oral Glucose Lowering Drugs
WHO: World Health Organization
